# Enhancement of Catalytic Activity of Reduced Graphene Oxide Via Transition Metal Doping Strategy

**DOI:** 10.1186/s11671-017-2196-4

**Published:** 2017-06-24

**Authors:** Hangil Lee, Jung A Hong

**Affiliations:** Department of Chemistry, Sookmyung Women’s University, Seoul, 04310 Republic of Korea

**Keywords:** Catalytic oxidation, Transition metal-doped rGO, HRPES, SEM, Electrochemical measurement

## Abstract

**Abstract:**

To compare the catalytic oxidation activities of reduced graphene oxide (rGO) and rGO samples doped with five different transition metals (TM-rGO), we determine their effects on the oxidation of *L*-cysteine (Cys) in aqueous solution by performing electrochemistry (EC) measurements and on the photocatalytic oxidation of Cys by using high-resolution photoemission spectroscopy (HRPES) under UV illumination. Our results show that Cr-, Fe-, and Co-doped rGO with 3+ charge states (stable oxide forms: Cr^3+^, Fe^3+^, and Co^3+^) exhibit enhanced catalytic activities that are due to the charge states of the doped metal ions as we compare them with Cr-, Fe-, and Co-doped rGO with 2+ charge states.

**Graphical Abstract:**

The SEM images and the corresponding EC measurements for (a) Cr3+, (b) Fe3+, and (c) Co3+ doped rGO
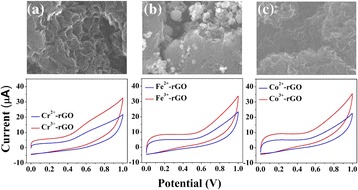

## Background

Recently, carbon-based materials such as reduced graphene oxide (rGO), graphene oxide (GO), and graphene are being more frequently used for various applications such as redox catalysts or fuel cells because they can be easily engineered by introducing functional groups [1–4]. Among them, rGO is an extensively utilized support for metal-based catalysts and can be used on its own as an oxidative catalyst because it has a high surface area, it exhibits high charge carrier mobility and electrochemical stability, and its surface can be readily engineered by introducing functional groups. In other words, rGO can also be used as an efficient metal-free catalyst for catalytic reactions because it can act as ideal components as we insert various dopants into rGO [5–11]. For these purposes, many research groups have tried to enhance the catalytic properties as they inserted various metals or molecules into rGO and reported the enhanced catalytic activities [12–20].

However, the catalytic effects of rGO can be enhanced with doping; one such approach is to insert cations or anions, but we need to be mindful of the costs of the fabricated catalyst as well as its efficiency [21–24]. Hence, we have been pursuing a strategy of doping rGO with transition metals (TM) to enhance its catalytic activity. For this purpose, in this study, we insert various transition metal ions (TM^+^) into rGO, and then systematically compare the catalytic activities of these rGO samples doped with transition metals (TM-rGO).

We evaluate the catalytic effects of these TM-rGO on the oxidation of *L*-cysteine (Cys) in aqueous solution by performing electrochemistry (EC) measurements and on the photocatalytic oxidation of Cys by using high-resolution photoemission spectroscopy (HRPES) under UV illumination. Cys can play an important role in the biological activity of proteins, so its detection is important in the fields of biology and diagnostics; the detection of Cys can be achieved by monitoring its oxidation reaction with electrochemical measurements [25, 26].

Here, we use a catalytic oxidation and an electrochemical oxidation of Cys to demonstrate a possible application of the synthesized TM-rGO as a catalyst for the sensitive detection of Cys. Hence, we finally compare the electronic properties and catalytic oxidation activities of the five TM-rGO samples with those of rGO by using scanning electron microscopy (SEM), Raman spectroscopy, EC measurements, and HRPES.

## Methods

### Preparation of the Precursor Solutions

We prepare each precursor solution with a one-pot synthesis. The desired amounts (wt % with respect to rGO (TM/(TM + rGO))) of the transition metal dopants are added in the form TM(NO_3_)_*x*_∙*n*H_2_O (metal nitrate *n*-hydrate). Cr(NO_3_)_3_·9H_2_O (99%), Mn(NO_3_)_2_·*x*H_2_O (98%), Fe(NO_3_)_3_·9H_2_O (98%), Co(NO_3_)_3_·3H_2_O (≥98%), Ni(NO_3_)_2_·6H_2_O (≥98%), FeCl_2_·4H_2_O (99%), CrCl_2_·4H_2_O (95%), and CoCl_2_·6H_2_O (99%) are purchased from Sigma-Aldrich and used as the dopants. Cys (Sigma-Aldrich, 99% purity) and Nafion (Sigma-Aldrich, 5 wt % in a low-molecular-weight aliphatic alcohol and water) are purchased from Sigma-Aldrich.

### Preparation of the Dispersed TM-rGO Solutions

**Fig. 1:**
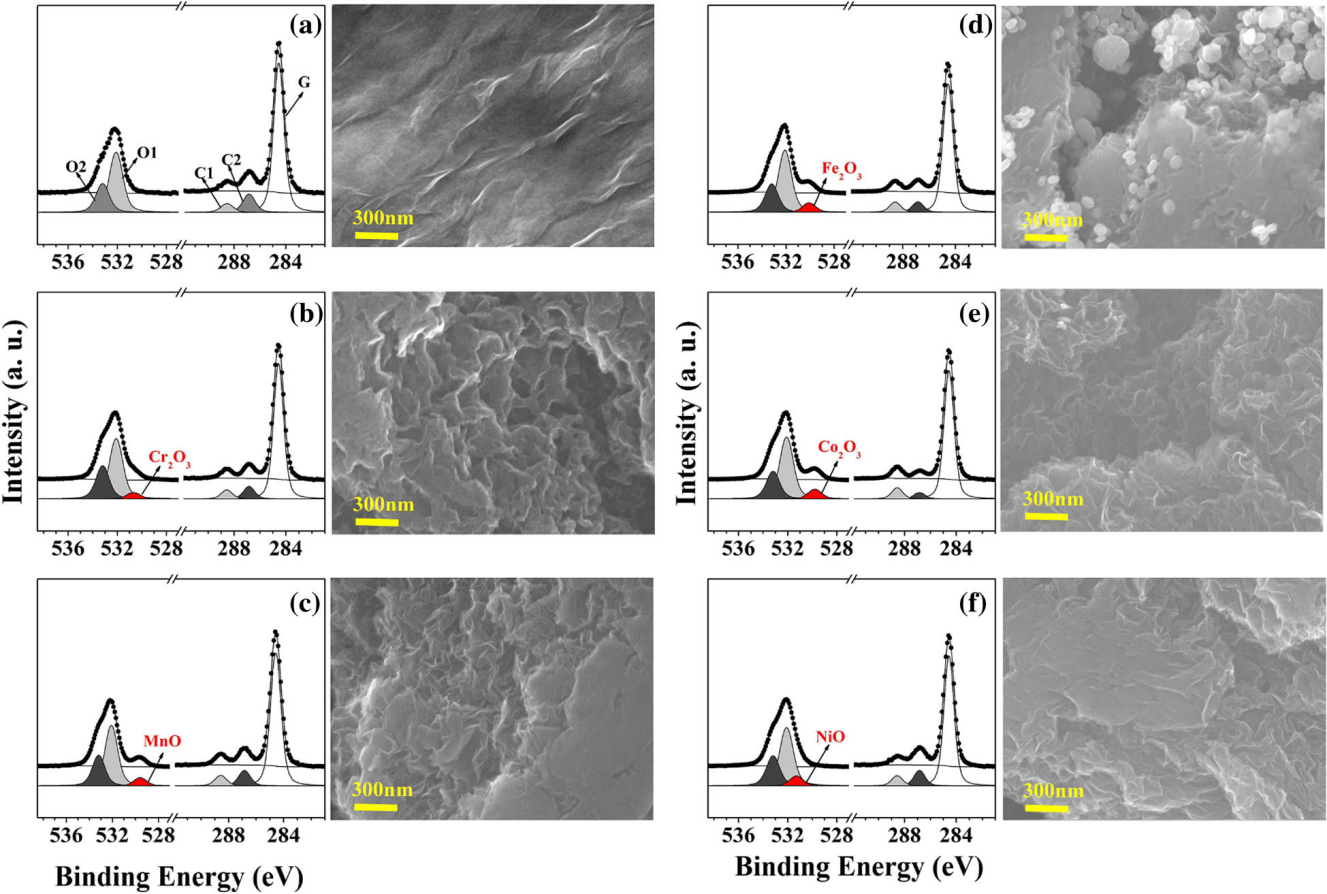
The HRPES spectra of monodisperse 5 mol % TM-rGO: **a** rGO, **b** Cr-rGO, **c** Mn-rGO, **d** Fe-rGO, **e** Co-rGO, and **f** Ni-rGO and the corresponding SEM images, respectively

rGO is obtained from GO by using Hummers’ method, which are carried out by adding hydrazine (1.0 mL) into a dispersion of GO (50 mg of GO in 100 mL of water) [27, 28]. After sonication for 1 h and stirring for 24 h at 60 °C, the fabricated rGO is characterized with C 1*s* core-level spectroscopy (see Fig. 1). Each of the five transition metal dopants is added to a rGO solution in an oil bath at 80 °C with stirring until the solution becomes transparent (approximately 20 min). The solutions are transferred to Teflon-lined autoclaves and then sealed and heated at 220 °C for 7 h in a convection oven. The resulting TM-rGO (Cr-rGO, Mn-rGO, Fe-rGO, Co-rGO, and Ni-rGO) samples are filtered and washed with double distilled water (DDW) to remove any residue.

### Fabrication of TM-rGO-Nafion-Modified GCEs and the Cys Oxidation Electrochemical Measurements

The electrochemical oxidation of Cys in the presence of glassy carbon electrodes (GCEs) modified with TM-rGO is investigated. For each TM, 4.0 mg of TM-rGO is dispersed into 2.0 mL of DDW containing 50 μL Nafion, and then mixed by using an ultrasonic processor for 5 min to obtain a homogeneous TM-rGO-Nafion mixture. Then, 20 μL of each mixture is deposited on a GCE and dried at 80 °C in a pre-heated oven for 30 min. Cyclic voltammetry (CV) is performed for each TM-rGO-Nafion-modified GCE in a 0.015 M Cys solution in PBS.

### Characterizations

Scanning electron microscopy (SEM) images of the samples are obtained by using a field-emission scanning electron microscope (JEOL JSM-7600F) operated at an acceleration voltage of 10 kV. Raman spectra are recorded by using a spectrometer (Horiba, ARAMIS) with an Ar^+^ ion CW (514.5 nm) laser. HRPES experiments are performed at the 8A2 beamline at the Pohang Accelerator Laboratory (PAL), which is equipped with an electron analyzer (SES100, Gamma Data Scienta). The S 2*p*, C 1*s*, and O 1*s* core-level spectra are obtained by using the photon energy 230, 340, and 580 eV, respectively. The electrochemical experiments are performed by using a CHI617B potentiostat (CH Instruments, Austin, TX) with a three-electrode cell placed in a Faraday cage. A GCE with a diameter of 2 mm is used as the working electrode, a Pt wire with a diameter of 1 mm is used as the counter electrode, and the reference electrode is Ag/AgCl (3 M KCl).

## Results and Discussion

First, we use HRPES to compare the electronic structures of the samples and to determine their carbon contents. The C 1*s* core-level spectra of rGO (Fig. 1a) and the TM-rGO samples (Fig. 1b~f) contain three deconvoluted peaks. The peak with the binding energy of 284.5 eV (G) is assigned to a C–C bond with *sp* ^2^ character. The other peaks are assigned to carbonyl groups (–C=O, 288.5 eV: C1) and hydroxyl groups (–C–OH, 286.7 eV: C2) [8]. We also acquire O 1*s* core-level spectra of rGO and the five TM-rGO samples. The core-level spectra indicate the presence of two typical functional groups in rGO: carbonyl and hydroxyl groups. Based on the relative electronegativity of these groups, the two –OH (hydroxyl group at 533.1 eV: marked as O2 peak) and –C=O (carbonyl group at 532.0 eV: marked as O1 peak) [29]. In addition, we also find O 1*s* core-level spectra induced the doped transition metal, which can change into the stable oxide forms (Cr_2_O_3_, MnO, Fe_2_O_3_, Co_2_O_3_, and NiO) [30–35].

SEM is used to characterize the morphologies of rGO and the five TM-rGO samples. The rGO sample of Fig. 1a consists of bundles, as found in previous results [22]. The SEM images of the five TM-rGO nanoparticles in Fig. 1b–f reveal structural features that are different to those of rGO: irregular shapes that have a layer-like structure with surface wrinkles due to scrolling, crumpling, and random aggregation to form disordered solids are present. In other words, we can clearly confirm the surface morphological change of metal-doped rGO being distinguishable from that of rGO as we clarify the increments of number of wrinkle on the surface. We believe that these are closely related to the catalytic active sites of metal-doped rGO. In other words, as we doped transition metals into rGO, we confirmed surface morphological changes of the number of wrinkle on surface, correlated with the increment of the active site, increasing and finally enhancing catalytic reaction on TM-rGO. As a result, we insist that these morphological changes of TM-rGO induced the doped transition metal (Cr, Mn, Fe, Co, and Ni) and can act as nucleation sites.

Next, we test the catalytic activities of these materials in the oxidation of Cys in aqueous solution by performing EC measurements and in the photocatalytic oxidation of Cys by using HRPES.

### Electrochemical Redox Reaction in the Aqueous Phase

**Fig. 2:**
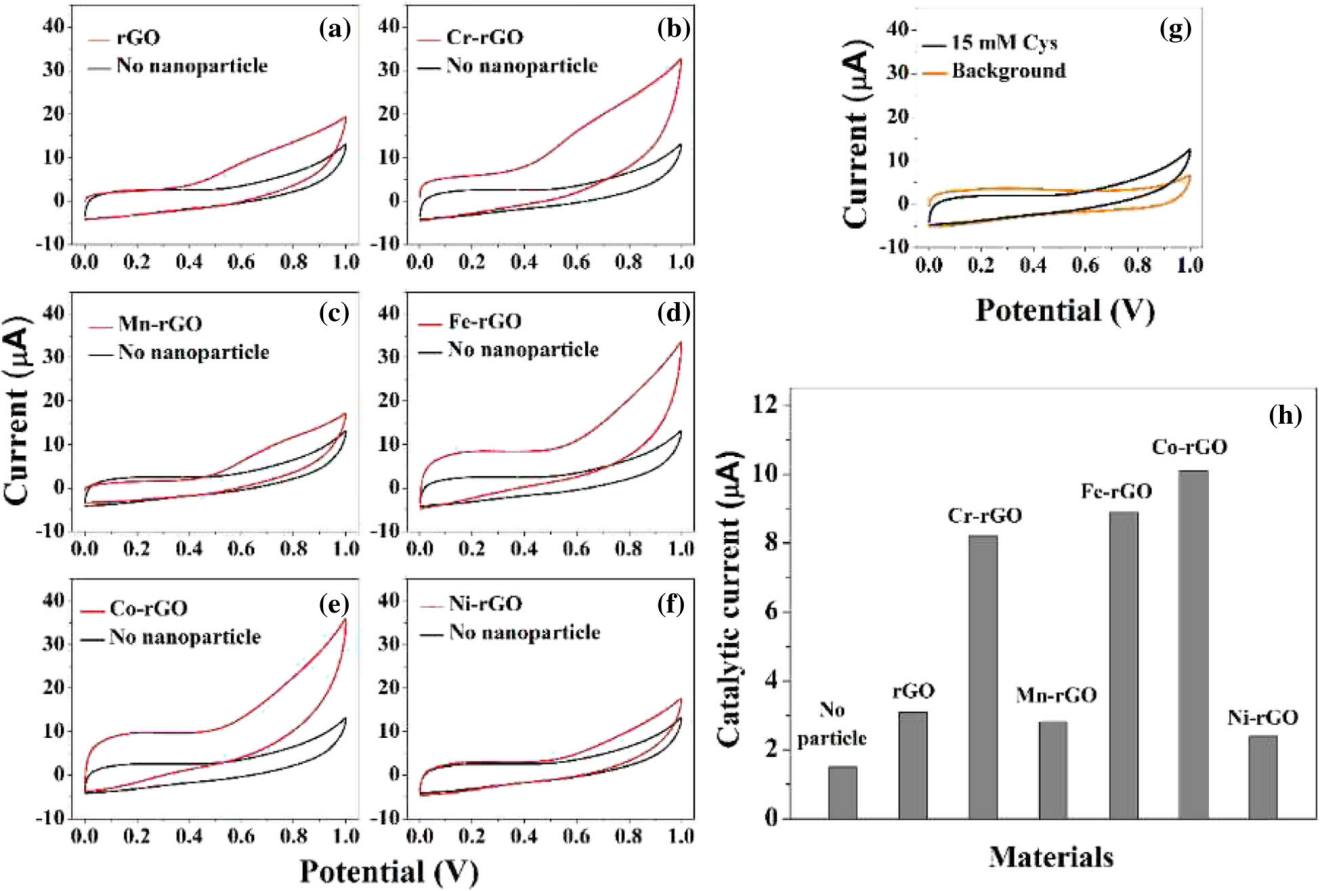
**a**–**f** CV (at a scan rate of 50 mV/s) of 0.015 M Cys solutions in PBS at bare GCEs (*black lines*) and at GCEs modified with 5 wt% **a** rGO, **b** Cr-rGO, **c** Mn-rGO, **d** Fe-rGO, **e** Co-rGO, and **f** Ni-rGO (*red lines*). **g** The oxidation currents observed at the bare GCEs are sluggish because of the intrinsically slow oxidation of Cys. **h** Currents resulting from the electrochemical oxidation of Cys in the presence of pristine rGO and the five TM-rGO catalysts

CV is performed in 0.015 M Cys solutions in PBS with various types of GCEs irradiated with 365-nm wavelength UV light. As shown in Fig. 2g, sluggish oxidation currents are observed at the bare GCEs because of the intrinsically slow oxidation of Cys. GCEs modified with rGO and TM-rGO-Nafion catalysts are also fabricated and tested; the results are shown in Fig. 2. The currents associated with the oxidation of Cys were 8.2 (±1.3) μA, 8.9 (±1.7) μA, and 10.1 (±1.6) μA for the GCEs modified with Cr-rGO, Fe-rGO, and Co-rGO nanoparticles, respectively; these values are significantly greater (i.e., 3.9, 4.1, and 5.4 times greater) than the current for the bare GCE, 2.0 μA (Fig. 2h). In contrast, the currents generated for pristine rGO, Mn-rGO, and Ni-rGO were only 1.5 (±0.4) μA, 2.7 (±0.8) μA, and 2.9 (±0.7) μA, respectively, which are slightly (1.3, 1.7, and 1.8 times) but not substantially greater than that for the bare electrode. These results for the catalysis of oxidation reactions reveal the importance of the type of metal doped into rGO, even when using 5 wt% of the doped transition metal, and specifically indicate that Cr-rGO, Fe-rGO, and Co-rGO are good catalysts for the oxidation of Cys.

### The Catalytic Oxidation of Cys

**Fig. 3:**
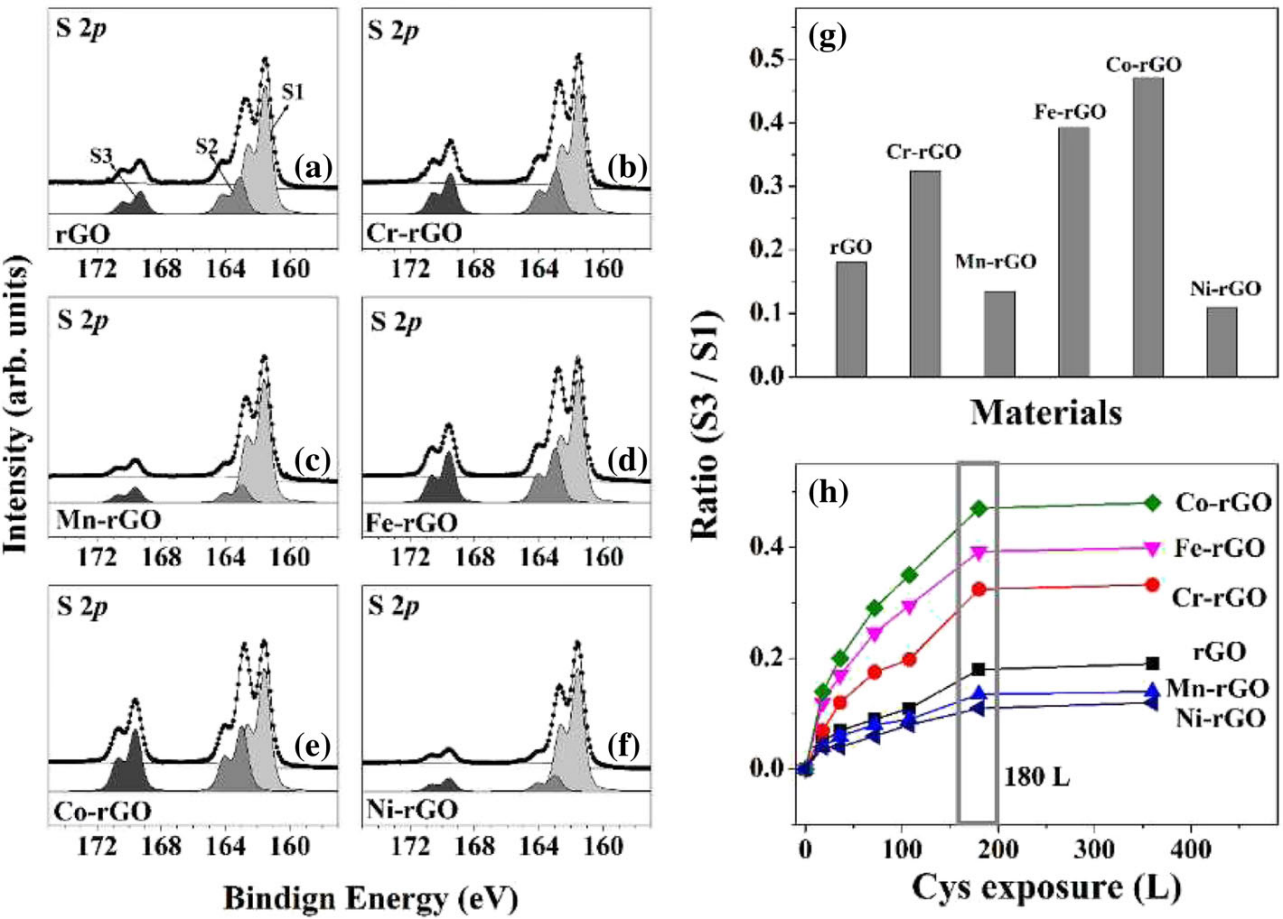
HRPES S 2*p* core-level spectra of the products of the photocatalytic oxidations of Cys (180 L) carried out in the presence of 5 wt % **a** rGO, **b** Cr-rGO, **c** Mn-rGO, **d** Fe-rGO, **e** Co-rGO, and **f** Ni-rGO (*left panel*). **g** S3-to-S1 ratios after 180 L of Cys exposure. **h** Ratios of the intensities of S3 and S1 for rGO and the five types of TM-rGO, resulting from various exposures of Cys to 365-nm wavelength UV light (*right panel*)

We next determine the direct catalytic activities of the TM-rGO samples in the oxidation of Cys molecules. The S 2*p* core-level spectra of pristine rGO and 5 wt% TM-rGO are obtained with HRPES after 180 L of Cys exposure in the presence of oxygen under 365-nm UV light illumination (see Figs. 3a–f). As shown in Fig. 3h, the ratio of the intensities of S3 and S1 does not vary for exposures greater than 180 L (the saturation coverage in our system). Hence, we perform oxidations with exposures to 180 L Cys, as shown in Fig. 3g. These spectra contain three distinct 2*p*_3/2_ peaks at 161.5, 162.9, and 168.6 eV, which are assigned to S1 (the C-SH unbounded state), S2 (the bound state), and S3 (sulfonic acid (SO_3_H)), respectively. It is well known that sulfonic acid is an oxidation product of thiol groups [36]. Hence, we can monitor the oxidation of Cys by measuring the ratio of the intensities of peaks S1 and S3. Figures 3a–f confirm that Cr-rGO, Fe-rGO, and Co-rGO act as effective photocatalysts. The ratios of the intensities are 0.18, 0.324, 0.135, 0.47, 0.392, and 0.11 for rGO, Cr-rGO, Mn-rGO, Fe-rGO, Co-rGO, and Ni-rGO, respectively, i.e., the ratios of Cr-rGO, Fe-rGO, and Co-rGO are larger than that of rGO and those of the other TM-rGO (Mn-rGO and Ni-rGO) (see Fig. 3(g)), and these results are closely correlated with the EC results.

Thus, two quite different catalytic measurements—the rate of electrochemical oxidation of Cys in aqueous solution and the rate of catalytic oxidation under ultra-high vacuum conditions—find the same trends in the catalytic activities of the five TM-rGO samples. This similarity arises even though the measurement conditions (in aqueous and under ultra-high vacuum conditions) are very different. Thus, the effects of the doping metal on the catalytic activity of rGO are independent of the environmental conditions.

**Fig. 4:**
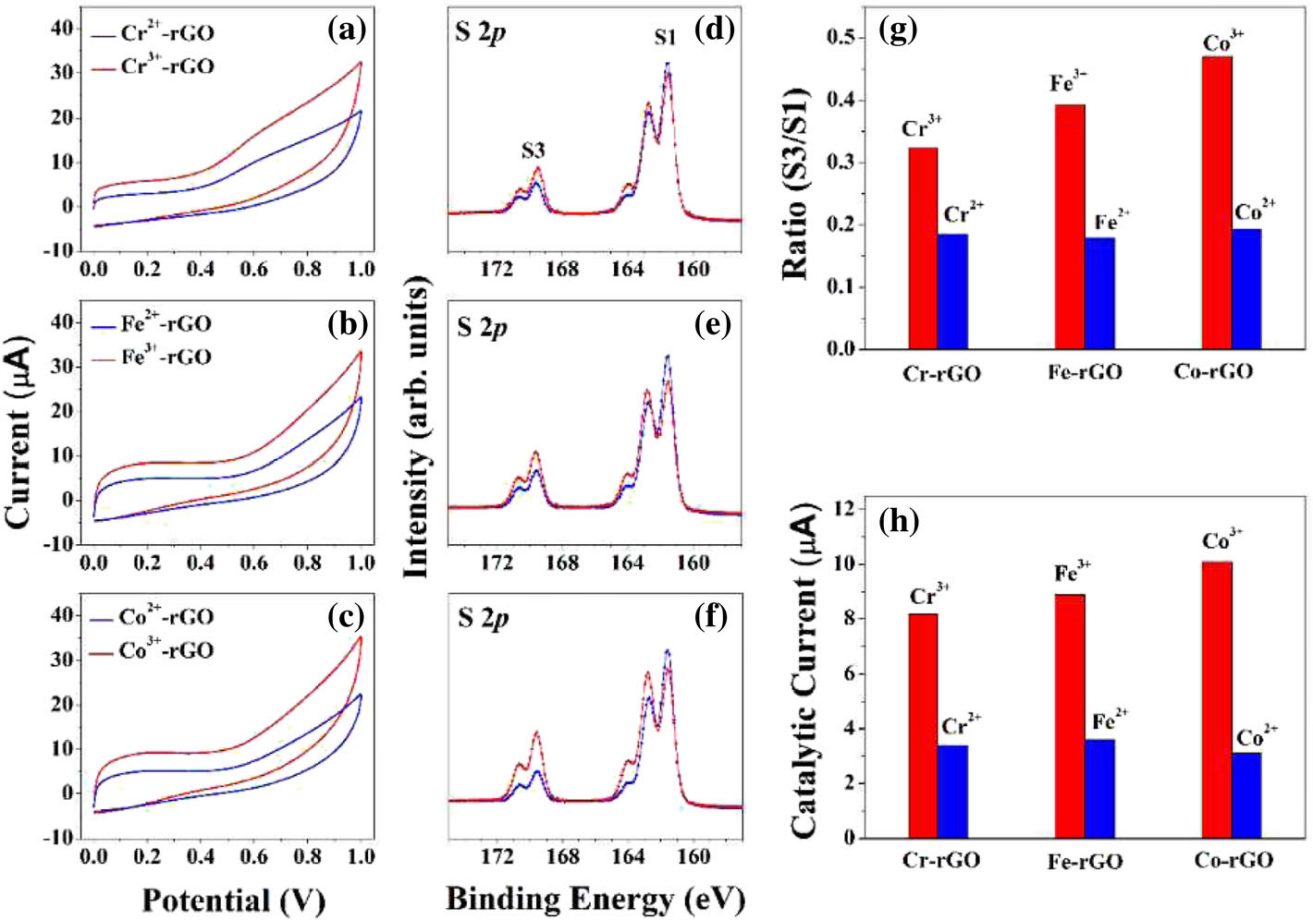
CV results at a scan rate of 50 mV/s for 0.015 M Cys solutions in PBS at GCEs modified with 5 wt% **a** Cr-rGO, **b** Fe-rGO, and **c** Co-rGO. HRPES S 2*p* core-level spectra of the products of the photocatalytic oxidations of Cys (after 180 L exposure) carried out in the presence (*red lines*: TM^3+^ and *blue lines*: TM^2+^) of 5 wt % **d** Cr-rGO, **e** Fe-rGO, and **f** Co-rGO. **g** Values of the S3-to-S1 ratio after 180 L Cys exposure. **h** Catalytic currents resulting from the electrochemical oxidations of Cys in the presence of the various types of pristine rGO and the three TM-rGO (*red lines*: TM^3+^ and *blue lines*: TM^2+^)

Our characterizations and spectral analyses have shown that Cr-, Fe-, and Co-doped rGO exhibit higher catalytic efficiencies than rGO, Mn-rGO, and Ni-rGO. There are two critical factors that influence the catalytic activities of the TM-rGO samples: the charge state, the surface morphology, and the formation of stable oxides on rGO. First, we investigated the effects of the electronic charge state by carrying out HRPES measurements. Figure 1a~f confirm that the Cr, Fe, and Co transition metal ions have TM^3+^ charge states whereas Mn and Ni have TM^2+^ charge states. Therefore, there is a correlation between the electron charge state of the TM-rGO dopant and its catalytic activity. Second, we assessed whether the stable oxides formed on rGO can enhance the catalytic activities of the samples. We compared the catalytic activities of Cr-, Fe-, and Co-rGO (+2 charge state) with those of Cr-, Fe-, and Co-rGO (+3 charge state) by performing EC measurements and photocatalytic oxidations of Cys; the results are shown in Fig. 4.

We perform CV measurements and photocatalytic oxidations of Cys in the presence of Cr-, Fe-, and Co-rGO with +2 charge states. This comparison of the catalytic efficiencies of +3 and +2 charge states confirms the role of stable oxides on rGO. Figures 4a–c show that the currents associated with the oxidation of Cys were 8.2 (±1.3) μA, 8.9 (±1.7) μA, and 10.1 (±1.6) μA when using the GCEs modified with Cr^3+^-rGO, Fe^3+^-rGO, and Co^3+^-rGO respectively, whereas they were only 3.4 (±0.7) μA, 3.6 (±0.8) μA, and 3.1 (±0.6) μA when using the GCEs modified with Cr^2+^-rGO, Fe^2+^-rGO, and Co^2+^-rGO, respectively.

We also determine the direct catalytic activities of the TM-rGO samples in the oxidation of Cys molecules. The S 2*p* core-level spectra of pristine rGO and 5 wt% TM-rGO are obtained with HRPES after 180 L of Cys exposure in the presence of oxygen under 365-nm UV light illumination (see Fig. 4d–f). The ratios of the intensities are 0.324, 0.392, and 0.47 for the +3 charge state Cr-rGO, Fe-rGO, and Co-rGO samples, respectively, whereas the ratios are only 0.184, 0.179, and 0.193 for the Cr-rGO, Fe-rGO, and Co-rGO samples with a +2 charge state, respectively. As shown in Fig. 3g, h, the ratios of Cr-rGO, Fe-rGO, and Co-rGO with a +3 charge state are higher than those of Cr-rGO, Fe-rGO, and Co-rGO with a +2 charge state, which indicates that our expectation is correct. These results reveal the importance of the formation of stable oxides on rGO to the catalysis of oxidation reactions, which is achieved by using 5 wt% of the doped transition metal, and demonstrated that Cr-rGO, Fe-rGO, and Co-rGO with +3 charge states are good catalysts for the oxidation of Cys.

Therefore, we conclude that the electronic charge state of the doped transition metal ion affects the enhancement of the catalytic activity. In particular, doping rGO with Cr, Fe, or Co with a +3 charge state yields a higher increase in the catalytic oxidation activity than doping with +2 charge state transition metals.

## Conclusions

In summary, we have compared the catalytic activities of rGO and rGO samples doped with five transition metals by performing the oxidation of Cys in their presence; we monitor the reactions by using high-resolution photoemission spectroscopy (HRPES) under UHV condition and electrochemistry (EC) measurements in aqueous solution. Our spectral analyses demonstrate that Cr-, Fe-, and Co-doped rGO with 3+ charge states (Cr^3+^, Fe^3+^, and Co^3+^) exhibit enhanced catalytic activities, i.e., these metal ions increase the surface reactivity. In conclusion, we have confirmed that the stable metal oxide forms of Cr^3+^, Fe^3+^, and Co^3+^ (Cr_2_O_3_, Fe_2_O_3_, and Co_2_O_3_) can act as good catalysts for oxidation reactions.

## Abbreviations

HRPES: High-resolution photoemission spectroscopy
SEM: Scanning electron microscopy
